# Human Gut Microbiome Can Degrade the Sweetener Acesulfame K with Potential Damaging Effects in the Intestinal Barrier Function

**DOI:** 10.1021/acs.jafc.5c16498

**Published:** 2026-04-21

**Authors:** Alicia Bellanco, Cristina Yépez-Notario, Marta Lozano, M. Carmen Martínez-Cuesta, Teresa Requena

**Affiliations:** † Department of Food Biotechnology and Microbiology, Instituto de Investigación en Ciencias de la Alimentación (CIAL-CSIC), Nicolas Cabrera 9, 28049 Madrid, Spain; ‡ Department of Microbiology and Biochemistry of Dairy Products, Instituto de Productos Lácteos de Asturias (IPLA-CSIC), Francisco Pintado Fe 26, 33011 Oviedo, Spain

**Keywords:** acesulfame K, food additive, gut microbiome, children, butyrate, sulfamate

## Abstract

Acesulfame K (Ace-K) is a commonly consumed sweetener, although knowledge about the Ace-K-gut microbiota interaction remains limited. This study evaluates dose-dependent effects of Ace-K on metataxonomics, metagenomics, and metabolic activity of children gut microbiota developed in a dynamic gut simulator. An Ace-K-dose dependent increase in *Anaerostipes*, *Coprococcus*, *Subdoligranulum*, *Blautia*, *Sutterella wadsworthensis*, *Alistipes,* and *Bacteroides thetaiotaomicron* was observed. Butyrate showed a dose–response increase that correlated with Ace-K consumption, suggesting its microbial metabolism. Increasing bacterial taxa showed sulfatase and amidase activities potentially capable of degrading Ace-K, releasing sulfamate and acetoacetate, which species such as *Anaerostipes hadrus* and *Intestinimonas* can metabolize to produce butyrate via the butanoyl-CoA pathway. Furthermore, the Ace-K-microbiome interaction led to a dose-dependent decrease in Caco-2 epithelial integrity, possibly due to the release of sulfated metabolites. This study provides evidence of the potential risk of Ace-K consumption based on its metabolism by the human gut microbiome.

## Graphical abstract

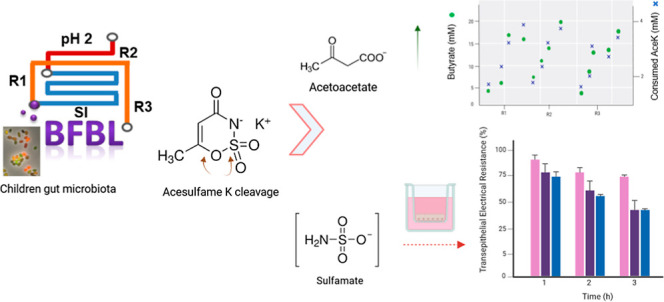

## Introduction

Reducing the intake of free sugars added to foods and beverages has been a public health priority during the last decades, including the World Health Organization (WHO) suggestion to reduce the consumption of free sugars to below 5% of total energy intake in order to prevent excessive weight gain, diet-related diseases, caries, and noncommunicable diseases in both adults and children.^1^ An increasing approach to lowering added sugar intake has been the replacement with nonsugar sweeteners (NSS) and sweetness enhancers.^2^ However, controversial opinions exist regarding NSS benefits in persons with obesity or prediabetes risks,^3,4^ including the WHO conditional recommendation that NSS should not be used as a means of achieving weight control or reducing the risk of noncommunicable diseases,^1^ as well as the opposing concerns suggesting the re-evaluation of this WHO recommendation.^5^

The major global intake of NSS corresponds to aspartame, acesulfame K (Ace-K), saccharin, sucralose, cyclamate, and thaumatin,^6^ being Ace-K one of the sweeteners most frequently used (e.g., 48.2% of total NSS consumed in Spanish foods^7^ and globally).^8,9^ Children and toddlers are among the population groups with higher NSS exposure levels,^10^ soft drinks being the main source for most sweeteners^11^ with Ace-K high-level exposures ranging up to 29 mg/kg body weight/day in children.^12^

Ace-K and other NSS are excreted primarily in the urine^13^ and evidence has increased about their regular detection as environmental pollutants due to their extensive use, persistence, and ubiquitous occurrence in various ecosystems.^14,15^ In fact, Ace-K has been used as a marker for domestic wastewater contamination in natural waters.^16^ However, the Ace-K persistence in wastewater seems to be changing due to increased evidence that Ace-K biodegradation is emerging^17^ likely due to microbial adaptation to metabolize it as a novel carbon source.^18^ The bacterial strains described so far to grow with acesulfame as sole carbon and energy source belong to the genera *Bosea*, *Chelatococcus,* and *Shinella*.^18,19^ Acesulfame metabolism has been experimentally verified using recombinant strains expressing sulfatase- and amidase-encoding genes.^20^ Additionally, exposure to artificial sweeteners such as Ace-K that contains the functional group of the sulfonamide antibiotics brings concerns about potential antimicrobial effects and the risk to contribute to the development of antimicrobial resistance in bacteria.^21^

The effective intestinal absorption of Ace-K might suggest inertness toward the gut microbiota;^22^ however, studies in mice^23–25^ and humans^26,27^ show arguable findings that should require further studies. The potential risk associated with the gut microbiota ability to metabolize Ace-K has not been addressed, considering that the compound can become biodegradable as a carbon source,^18^ releasing sulfamic acid as an end product.^19^ Given the potential high NSS exposure levels observed in children and the scarcity of studies comparing a range of increasing doses, we have evaluated dose-dependent effects of Ace-K intake on the bacterial composition (sequencing of 16S rRNA gene amplicons) and function (shotgun metagenomics) and the metabolic activity (degradation of Ace-K and formation of short-chain fatty acids) of children’s gut microbiota developed in a dynamic simulator of the colonic microbiota (BFBL gut simulator). Evaluation of the effect on the intestinal barrier function was also performed with Caco-2 cell cultures. The study intends to provide scientific data that could be relevant in the risk assessment re-evaluation of Ace-K as a sweetener.

## Materials and Methods

### Gut Microbiota Inoculum

Faecal samples were donated by children aged 4 to 6 years. Privacy rights of volunteers have been observed, and samples were recruited after authorization from the parents who signed an informed consent of the protocol. The procedure was approved by the CSIC ethics committee (codes 181/2021, issued on November 3, 2021; and 051/2024, February 26, 2024). The use of human samples was carried out in accordance with the World Medical Association Declaration of Helsinki. Samples were transported in a zip-closed bag including an anaerobe-gas generation sachet (AnaeroGen, Oxoid) and stored at −80 °C. The inoculum was a pooled mix of samples from five individuals as described earlier.^28^

### BFBL Gut Simulator for the Dynamic Reproduction of Colonic Microbiota

The three reactors of the BFBL gut simulator (ascending colon: R1, pH 5.8; transverse colon: R2, pH 6.3; descending colon: R3, pH 6.8) were inoculated (1%) with the homogenized faecal pool and incubated overnight under static conditions at 37 °C in anaerobiosis with nutrient medium.^29^ Stabilization of the gut microbiota was achieved by feeding the small intestine (SI) three times a day for 1 week with nutritive medium (pH 2) mixed with pancreatic juice and bile salts.^30^ After digestion (2 h at 37 °C), the content of the SI was automatically transferred at a constant flow rate (5 mL/min) to the colonic reactors, whose volume, controlled by level sensors, was maintained at 200 mL (R1), 300 mL (R2), and 250 mL (R3). After the week of stabilization (W1),^25^ increasing doses (0.5, 1.5, 3, and 5 g/L) of Ace-K were administered 3 times daily (at 8 h intervals) for 1 week for each dose (W2, W3, W4, and W5, respectively) to evaluate dose–response effects. These doses are equivalent to 5, 15, 30, and 50 mg/kg/day for an estimated average weight of 18 kg for 4–6 year old children. The doses included the acceptable daily intake (ADI) of 15 mg/kg body weight allocated by the EFSA and FDA health authorities,^31^ and increasing concentrations, which included reported high-level exposures in children,^12^ to determine dose-dependent effects. An equivalent experiment was carried out without the addition of Ace-K during the same period of time (W1–W5), as a control (Ctrl). Samples were collected daily and centrifuged (10,000*g*, 4 °C, 10 min), and pellets and supernatants stored separately at −80 and −20 °C, respectively. Samples from the last 3 days of each stage were treated as triplicates.

### Metagenomics Based on the Sequence of 16S rRNA Gene Amplicons and Shotgun

DNA from the pellets was extracted using the commercial E.Z.N.A. bacterial DNA kit (Omega Biotek) and a FastPrep instrument (Bio 101 FastPrep FP120, Savant Instruments) for mechanical lysis. DNA samples were quantified using a Nanodrop (NanoDropH ND-1000 UV spectrophotometer, Nano-Drop Technologies), stored at −20 °C and shipped to Novogene (Germany). The V3–V4 region of the 16S rRNA gene was amplified using 341F (5′-CCTAYGGGRBGCASCAG-3′) and 806R (5′-GGA CTACNNGGGTATCTAAT-3′) primers. PCR products were sequenced on the Illumina paired-end platform to generate 250 bp paired-end raw reads. Sequences with a similarity of >97% were assigned to the same operational taxonomic unit (OTU), and the SILVA138 SSUrRNA database was used for taxonomic annotation.

Shotgun analysis was performed with the microbial pellets from the W5 last 3 days (5 g/L Ace-K and control) of R1 and R3 colonic reactors of the BFBL gut simulator since they are complementary in reproducing the child gut microbiota.^32^ Sequencing libraries were generated with fragmented genomic DNA, A-tailed, and ligated with full-length adapters for Illumina sequencing at Novogene. MEGAHIT software was used for metagenome assembly, MetaGeneMark to perform ORF prediction for scaftigs higher than 500 bp, and CD-HIT to obtain the nonredundant initial unigene catalogue. DIAMOND software was used to align unigenes with those in the functional databases KEGG, eggnog, CAZy, VFBD, PHI, and CARD.

### Analysis of Acesulfame K, Short-Chain Fatty Acids, and Ammonium

The concentration of Ace-K consumed during its supplementation to the BFBL gut simulator was analyzed in the supernatants by UV absorption spectra (from 200 to 300 nm) and A_225_ reading (maximum Ace-K absorbance). Calibration was carried out in an Ace-K range concentration of 0.1–1 mM.

SCFAs, from the 0.22 μm-filtered supernatants, were quantified using an HPLC system (Jasco) equipped with a UV-975 detector. SCFAs were separated using a Rezex ROA Organic Acids column (Phenomenex) at 50 °C and a mobile phase with a gradient of 0.005 M sulfuric acid in water at a flow rate of 0.6 mL/min. The elution profile was monitored at 210 nm, and the SCFA concentration (mM) was obtained through calibration curves of acetic, propionic, and butyric acids in the range concentration of 1–100 mM.

The ammonium content was determined by incubating the supernatants with Nessler’s reagent (Sigma-Aldrich) 5 min at room temperature as described earlier,^33^ whose absorbance (425 nm) was measured with a Varioskan Plate reader (Thermo Fisher Scientific). Ammonium quantification was performed using an ammonium chloride calibration curve in the concentration range of 0–20 mM.

### Analysis of Intestinal Epithelial Barrier Function In Vitro

The human colon adenocarcinoma cell line Caco-2 (HTB-37, ATCC, Manassas, VA, USA) was used as an *in vitro* intestinal epithelial model. Caco-2 cells were cultured in Dulbecco’s Modified Eagle’s Medium (DMEM) supplemented with 10% fetal bovine serum, 1% nonessential amino acids, and 1% penicillin–streptomycin in a humidified incubator (Binder GMbH) with a controlled atmosphere of 5% CO_2_. The medium was changed every other day, and cells were subcultured when they reached 80% confluence. The mentioned reagents were purchased from Biowest.

#### Analysis of Intestinal Cell Viability

Caco-2 cells were seeded in 96-well plates at a density of 10^4^ cells per well and incubated for 1 week with respective medium changes. Then, the cells were treated for 24 and 48 h with Ace-K in the range of increasing concentrations up to 20 g/L to obtain a survival curve. Supernatants from R2 (transverse colon, pH 6.3) of the BFBL gut simulator supplemented with Ace-K and sulfamic acid up to 10 mM were also tested. After this time, the treatment was removed and 100 μL of 3-(4,5-dimethylthiazol-2-yl) 2,5-diphenyltetrazolium bromide (MTT) (0.5 mg/mL) was added to each well and incubated at 37 °C for 3 h in dark. Then, MTT was removed, and 100 μL of a dimethyl sulfoxide and ethanol mixture (1:1) was added and incubated for 30 min at 37 °C in the dark under soft shake. Once the formazan crystals were solubilized, absorbance was measured on a Varioskan instrument at 570 nm. Cell viability was calculated as the percentage of live treated cells with respect to the untreated cells (0 g/L). Based on the cell viability values, the concentration at which 50% of cell growth was inhibited (IC_50_) was calculated using a nonlinear regression model using GraphPad Prism 8.0 (GraphPad Software).

#### Analysis of Epithelial Integrity and Paracellular Permeability

Caco-2 cells were seeded onto apical inserts of Transwell plates (12 mm Transwell with 0.4 μm Pore Polyester Membrane Insert, Corning) at a density of 5 × 10^4^ cells per well and incubated for the next 15 days until confluence and transepithelial electrical resistance (TEER) became stable, with respective medium changes. Cells were then incubated for 3 h with Ace-K at the concentrations administered to the BFBL simulator (0, 0.5, 1.5, 3, and 5 g/L) and with the microbial pellets and supernatants from R2 (transverse colon, pH 6.3) of the Ace-K-fed BFBL simulator (W1, W2, W3, W4, and W5). Cell monolayer integrity was assessed by measuring TEER with a voltimeter (EVOM3 Epithelial Volt/Ohm Meter, World Precision Instruments) at times 0, 1, 2, and 3 h. Afterward, the treatments were removed, and wells were washed with Hank’s Balanced Salt Solution (HBSS; Sigma-Aldrich). Then, 500 μL of the Lucifer Yellow (Sigma-Aldrich) fluorescent dye (50 μM) was added to the apical inserts, and 1.5 mL of HBSS was added to the basolateral wells and incubated for 1 h in the dark. Afterward, 100 μL was collected from the basolateral wells, and fluorescence was measured (ex: 485 nm, em: 520 nm) using a microplate reader (FLUOstar Optima, BMG Labtech). Epithelial integrity was calculated as the percentage of TEER with respect to 0 h time of each treatment. The Lucifer Yellow concentration (μM) was calculated using a standard curve. When testing Ace-K alone, no treatment (untreated cells) was used as a control (0 g/L). When BFBL samples were tested, pellets and supernatants from stabilization (W1) were used as controls.

### Statistical Analysis

All measurements were performed in triplicate. Microbial composition data were expressed as medians and 95% confidence interval (CI) of the relative abundance values of each OTU (%) calculated using GraphPad Prism. Alpha-diversity, metabolic activity, epithelial integrity, and paracellular permeability data were expressed as mean and standard error of the mean (SEM) of Shannon, Simpson, and Chao1 indexes, concentration (mM) of microbial metabolites, TEER (%), and concentration (μM) of Lucifer Yellow, respectively. One-way ANOVA was used to evaluate the differences between the different treatment groups in diversity indexes, SCFAs and ammonium, and epithelial cell assays. Spearman’s correlation (ρ) was used to analyze the correlation between Ace-K doses administered and changes in the OTUs abundance, and Pearson’s correlation (*r*) was used to analyze the correlation between doses administered and changes in the microbial metabolite concentration. Values of *p* ≤ 0.05 (*) and ≤0.01 (**) were considered statistically significant. Statistical analyses were performed using SPSS Statistics for Windows, version 29.0 (IBM Corporation).

## Results

### Effects of Acesulfame K on the Gut Microbiota Composition

Feeding increasing doses of Ace-K into the BFBL gut simulator, previously inoculated with a pooled children fecal microbiota, showed no changes in alpha-diversity when compared with the control at the same time intervals, according to the values of the Shannon, Simpson, and Chao 1 alpha-diversity indexes (Table S1). Therefore, Ace-K does not seem to have exerted a significant antimicrobial effect during the study, despite containing the antibiotic-like sulfonyl group of sulfamides.^34^

The taxonomic examination through the OTUs did show some differences upon feeding with Ace-K with respect to the control experiment. Belonging to the phylum Bacillota (syn. Firmicutes), a dose-dependent increase in the relative abundance of the genera *Anaerostipes* (Spearman ρ 0.777** in R1, ρ 0.734** R2, ρ 0.577* R3), *Subdoligranulum* (ρ 0.770** R1, ρ 0.829** R2, ρ 0.917** R3), and *Coprococcus* (ρ 0.644** R1, ρ 0.578* R2, ρ 0.666** R3) was observed. Likewise, the genus *Blautia* (Figure 1a) also increased its relative abundance in all three reactors in a dose-dependent manner from approximately 0.5% (W1) to 4.5% (W5) in R1, R2, and R3. At the species level, these changes were mainly attributed to the increase in the number of OTUs assigned to *Anaerostipes hadrus* (Figure 1b), which increased from 0.5% (W1) to approximately 2.5% (W4) in R1, from 0.2% (W1) to 1.3% (W4) in R2, and from 0.2% (W1) to 0.9% (W4) in R3, and *Coprococcus comes* (Figure 1d), which increased from 1.7% (W1) to 2.5% (W5) in R1 and from 1.5% and 1.1% (W1) in R2 and R3, respectively, to 2.0% (W5) in both R2 and R3.

**Figure 1:**
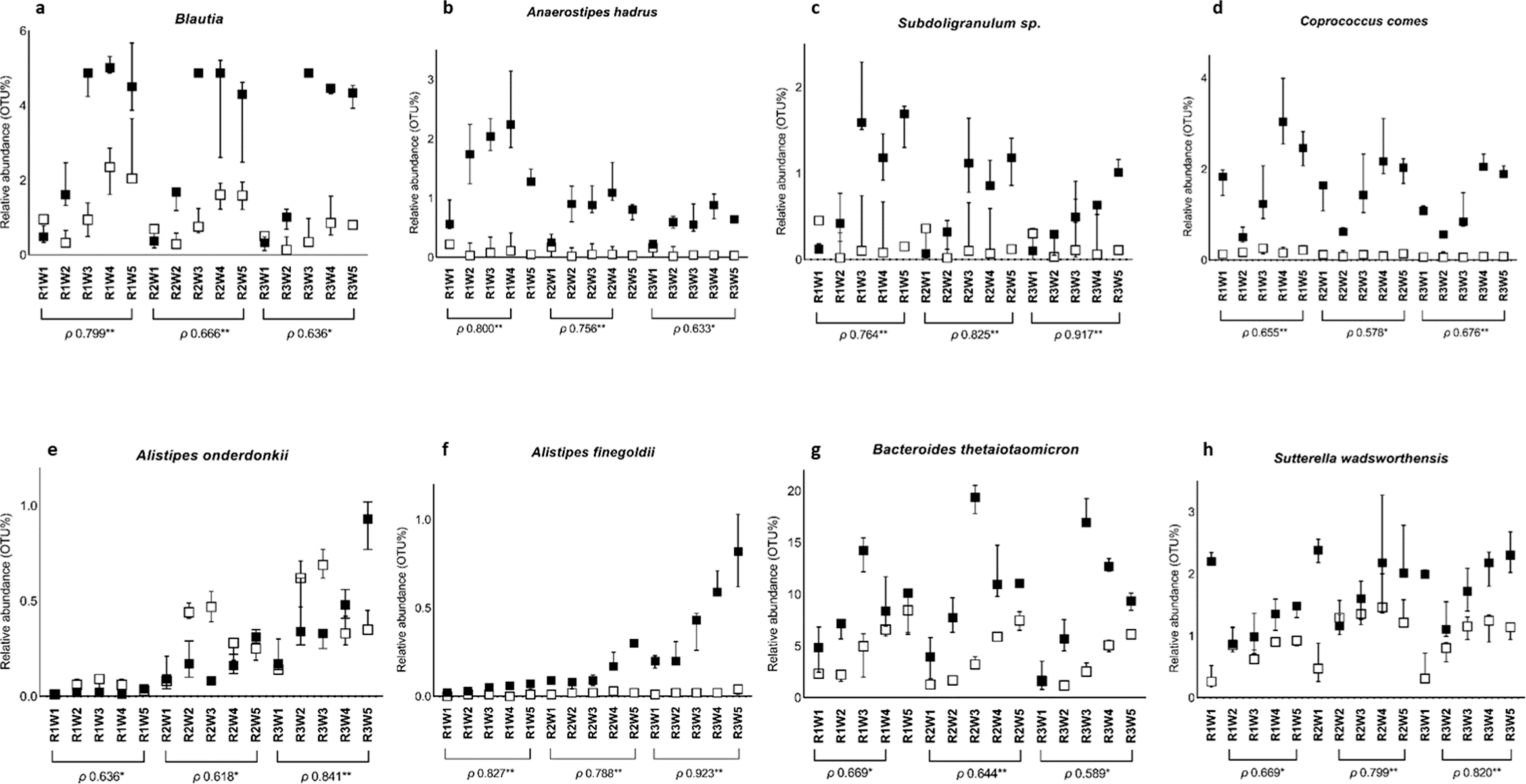
Relative abundance (median ±95% CI) of the genus (a) *Blautia* and the species (b) *A. hadrus*, (c) *Subdoligranulum* sp., (d) *Coprococcus comes*, (e) *Bacteroides thetaiotaomicron*, (f) *Alistipes onderdonkii*, (g) *Alistipes finegoldii,* and (h) *Sutterella wadsworthensis* in the three colonic reactors of the BFBL gut simulator (ascending colon: R1, pH 5.8; transverse colon: R2, pH 6.3; descending colon: R3, pH 6.8) supplemented with Ace-K (■) at ascending doses (0, 0.5, 1.5, 3, and 5 g/L; samples W1, W2, W3, W4, and W5, respectively) and the control (□) during the same period of time. Spearman’s Rho (ρ) indicates correlation between the relative abundance of the OTUs (%) and the administered doses of Ace-K. * and ** denote significant correlation (*p* < 0.05 and *p* < 0.01, respectively).

As for genera belonging to the phylum Bacteroidota (syn. Bacteroidetes), *Alistipes* increased showing a dose–response effect in all three colonic reactors (ρ 0.859** R1, ρ 0.775** R2, ρ 0.906** R3), whose increased abundance was mainly attributed to the increase in the number of OTUs assigned to the species *Alistipes onderdonkii* (Figure 1e) and *Alistipes finegoldii* (Figure 1f), especially in R3 from 0.2% (W1) to 0.85% (W5). Also, the species *Bacteroides thetaiotaomicron* (Figure 1g) increased its abundance in all three reactors in a dose-dependent manner, from 4.7%, 3.9%, and 1.5% (W1) in R1, R2, and R3, respectively, to 8.3% (W4) in R1, 11.4%, and 9.3% (W5) in R2 and R3, respectively, with a maximum abundance when 1.5 g/L Ace-K (W3) was administered to the BFBL gut simulator.

The abundance of *Sutterella wadsworthensis* (Figure 1h), belonging to the phylum Pseudomonadota (syn. Proteobacteria), increased showing a dose–response effect in all three reactors from the administration of the first dose (0.5 g/L, corresponding to W2 in the control) from 0.8% in R1 and 1.2% in R2 and R3 to 1.8%, 2.2%, and 2.3% (W5) in R1, R2, and R3, respectively.

### Effects of Acesulfame K on Gut Microbiota Metabolism

The butyric acid concentration increased in all three colonic reactors in a dose-dependent manner during the Ace-K feeding (Figure 2), in contrast to the stable values in the control experiment with mean values (mM) of 12.9 ± 0.7 (R1), 9.8 ± 0.4 (R2), and 2.8 ± 0.2 (R3) through the 5 weeks of study (Table S2). The highest butyric acid concentration during the Ace-K feeding reached values of 20.8 ± 1.3, 17.6 ± 0.6, and 13.5 ± 0.6 mM in R1, R2, and R3, respectively, with the administration of the highest dose of Ace-K (5 g/L, corresponding to W5). Similarly, the Ace-K consumed showed a dose-dependent increase that significantly correlated with the produced butyric acid (Figure 2).

**Figure 2:**
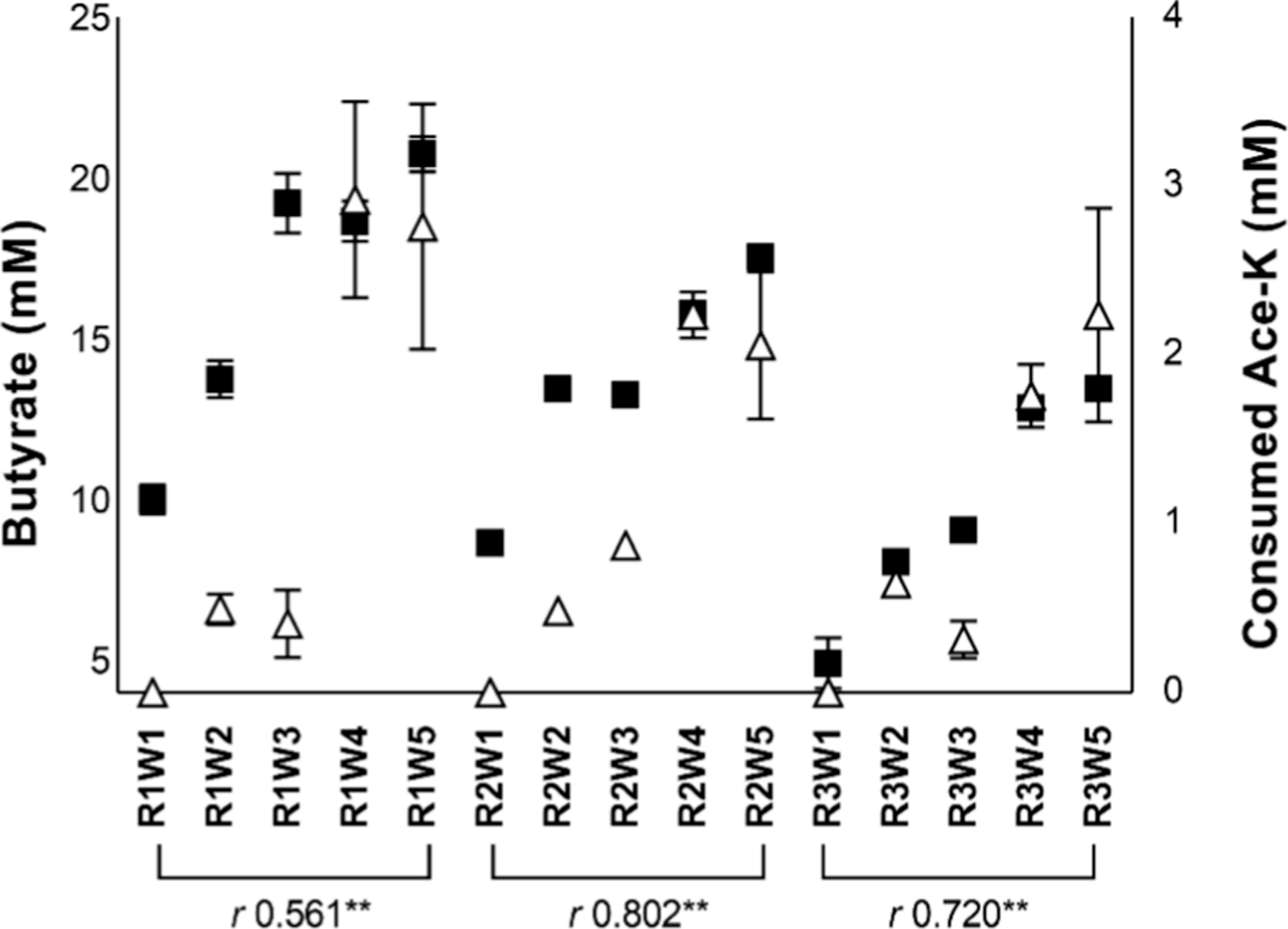
Concentration (mM) of butyrate (■) and consumed Ace-K (△) (mean ± SEM) in the three colonic reactors of the BFBL system (ascending colon: R1, pH 5.8; transverse colon: R2, pH 6.3; descending colon: R3, pH 6.8) supplemented with increasing doses of Ace-K (0, 2.5, 7.5, 15, and 25 mM; samples W1, W2, W3, W4, and W5, respectively). Pearson’s coefficient (*r*) indicates the correlation of the microbial produced butyrate and consumed Ace-K by the Ace-K-fed microbiota. **Denotes significant correlation (*p* < 0.01).

On the other hand, no significant differences were observed in any of the three colonic reactors with the concentration of propionic and acetic acids and ammonium when comparing samples from the BFBL gut simulator supplemented with Ace-K and the control experiment, with both following similar trends (Table S2).

### Metagenomics

#### Microbial Enzymes Related to Ace-K Metabolism

The findings regarding the correlation between Ace-K consumption and production of butyric acid could be related to the ability of the gut microbiome to partially degrade and utilize Ace-K as a carbon source. Thus, we evaluated with the use of metagenomic technology the relationship between microbial populations and potential enzymatic degradation of Ace-K. In fact, the shotgun analysis revealed that some species present in the reactors supplemented with Ace-K (microbiomes R1 and R3) possessed enzymatic functions involved in the microbial degradation of Ace-K, specifically sulfatases, amidases, and butyrate-acetoacetate CoA-transferase activities (Supporting Information File), as previously described.^19^

Arylsulfatases (EC 3.1.6.1 and 3.1.6.8) in the R1 and R3 microbiomes were represented mainly by taxons from the classes Bacteroidales and Eubacteriales, being more abundant in Ace-K samples than in the control (Supporting Information File). At the species level, the arylsulfatase EC 3.1.6.1 was mainly represented by *Barnesiella intestinihominis*, *Hungatella hathewayi,* and *Pusillibacter faecalis* (Figure 3). The amidase activity (EC 3.5.1.4) in R1 was identified in *Bilophila wadsworthia*, *Enterocloster lavalensis*, *Phascolarctobacterium faecium,* and *S. wadsworthensis*, while in R3, it was associated with *Anaerotruncus colihominis*, *Bifidobacterium longum*, *Eisenbergiella* sp., *Klebsiella michiganensis*, *Klebsiella* sp., *Raoultella* sp., *Rhodococcus erythropolis*, and *Rhodococcus* sp. Finally, butyrate-acetoacetate CoA-transferase activity (EC 2.8.3.9) was detected in *A. hadrus*, *Coprococcus catus*, *Eubacterium ramulus,* and *P. faecium* in R1, while it was associated with *Enterocloster clostridioformis*, *Intestinimonas butyriciproducens*, *Intestinimonas massiliensis*, *Lawsonibacter celer,* and *Pusillibacter faecalis* in R3. Relative abundance of the butyrate-producing enzyme was highest in the Ace-K sample from the R3 microbiome (Supporting Information File).

**Figure 3:**
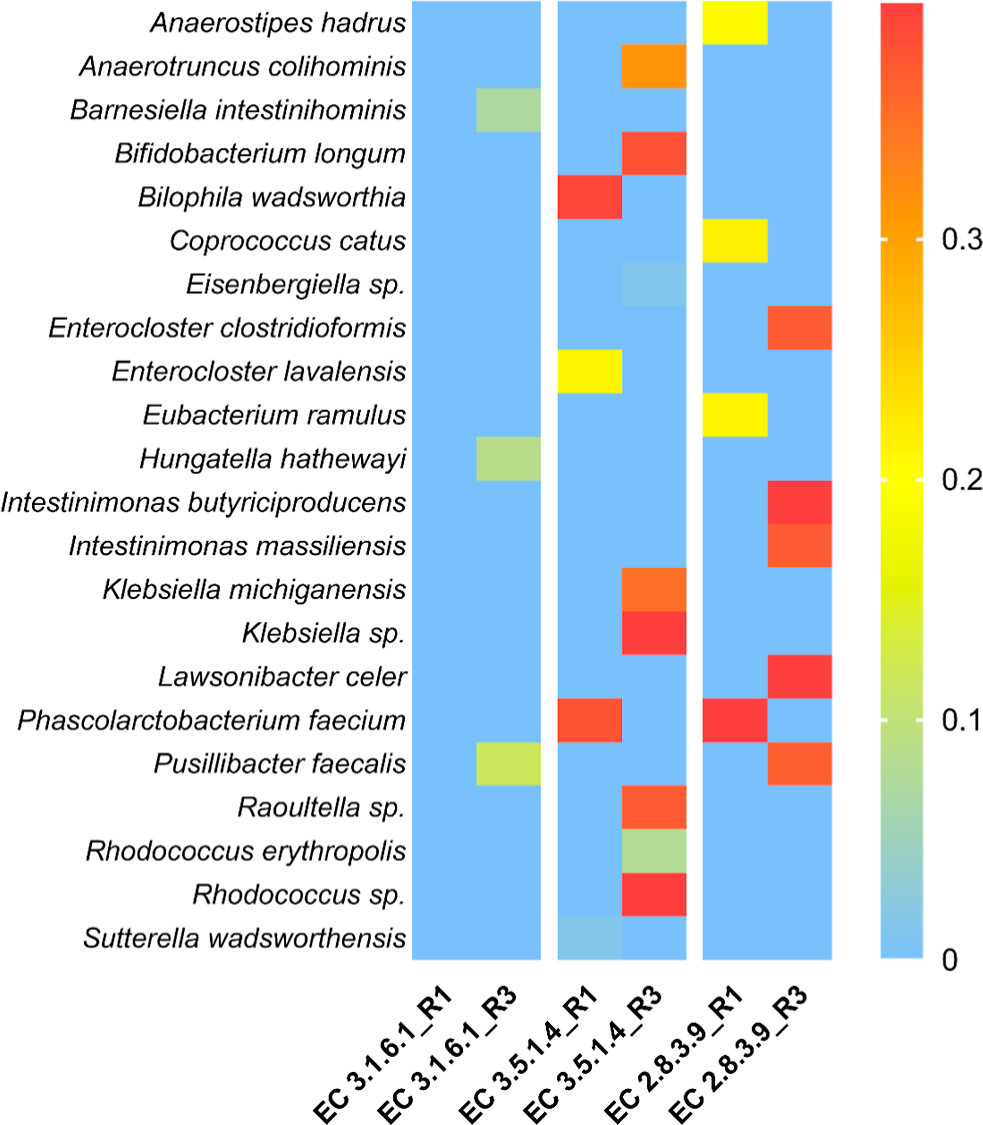
Heatmap based on the metagenomic analysis and representing the species that possess enzymatic activities potentially involved in the microbial degradation of Ace-K (EC 3.1.6.1, arylsulfatase; EC 3.5.1.4, amidase; and EC 2.8.3.9, butyrate-acetoacetate CoA-transferase) in the microbiome from reactors R1 (ascending colon, pH 5.8) and R3 (descending colon, pH 6.8), at the last week of Ace-K feeding (5 g/L; W5).

#### Effect of Acesulfame K on Gut Microbiota Antibiotic Resistance

The functional analysis of the unigene sequences from the Ace-K and control experiments at the end of the studies using the CARD database revealed no significant increases in the relative abundance of the main annotated genes conferring antibiotic resistance in the child gut microbiota treated with increased doses of Ace-K when compared with the control (Table S3). The most abundant antibiotic resistance genes observed were *van* (glycopeptide antibiotics), *adeF* (fluoroquinolone), and *tet* (tetracycline). In particular, the sulfonamide resistance gen *sul2* was not increased in the Ace-K metagenome (Table S3).

#### Effect of the Interaction of Intestinal Microbiome and Acesulfame K on the Intestinal Barrier Function

First, to differentiate the effects produced by Ace-K *per se* from those related to the interaction of Ace-K with the intestinal microbiome, the impact of Ace-K alone on the Caco-2 cells was assessed. Cell viability was evaluated after Ace-K treatment at a range of concentrations (0–20 g/L) to determine the IC_50_ value as well as epithelial integrity and paracellular permeability after Ace-K treatment at the same concentrations at which it was administered to the BFBL gut simulator. Cell viability values after 24 and 48 h incubation showed an IC_50_ value of 14.8 g/L at 24 h and 11.6 g/L at 48 h (Figure S1). These concentrations, which are about 3-fold higher than those administered to the BFBL gut simulator, could be considered not cytotoxic. On the other hand, supernatants from BFBL gut simulator (R2) supplemented with Ace-K (W4 and W5) decreased the cell viability by 25% and 37%, respectively (Figure S2).

The epithelial integrity after 1, 2, and 3 h of incubation with Ace-K (0.5, 1.5, 3, and 5 g/L) did not decrease, according to TEER values, which showed no difference with respect to the control (0 g/L) (Figure S3a). The paracellular permeability did not increase either after 3 h of incubation with Ace-K at the same concentrations, according to the concentration of Lucifer Yellow in the basolateral part of the inset, which showed no difference with respect to the control (0 g/L) (Figure S3b). Therefore, the doses of Ace-K administered to the BFBL gut simulator did not cause any effect on epithelial integrity nor on paracellular permeability.

Microbial pellets and supernatants from the Ace-K-fed BFBL simulator (W1, W2, W3, W4, and W5) were then tested. After incubating the pellets for 1, 2, and 3 h, no changes in epithelial integrity were observed at any time with respect to the stabilization period (W1) (Figure 4a). In contrast, after incubating the supernatants (Figure 4b), Caco-2 epithelial integrity decreased with respect to the stabilization period (W1) according to TEER values, which decreased (*p* < 0.05) after 1 h of incubation to 57% and 46% when supernatants from the highest doses of Ace-K feeding (3 and 5 g/L, corresponding to W4 and W5) were tested. The epithelial integrity followed some trend to stabilize over time, with TEER values of 77% and 69% being obtained for samples W4 and W5, respectively, after 3 h of incubation. At 3 h, however, the epithelial integrity was lower (*p* < 0.05) compared to the stabilization period (W1), starting at 1.5 g/L (W3, W4, and W5).

**Figure 4:**
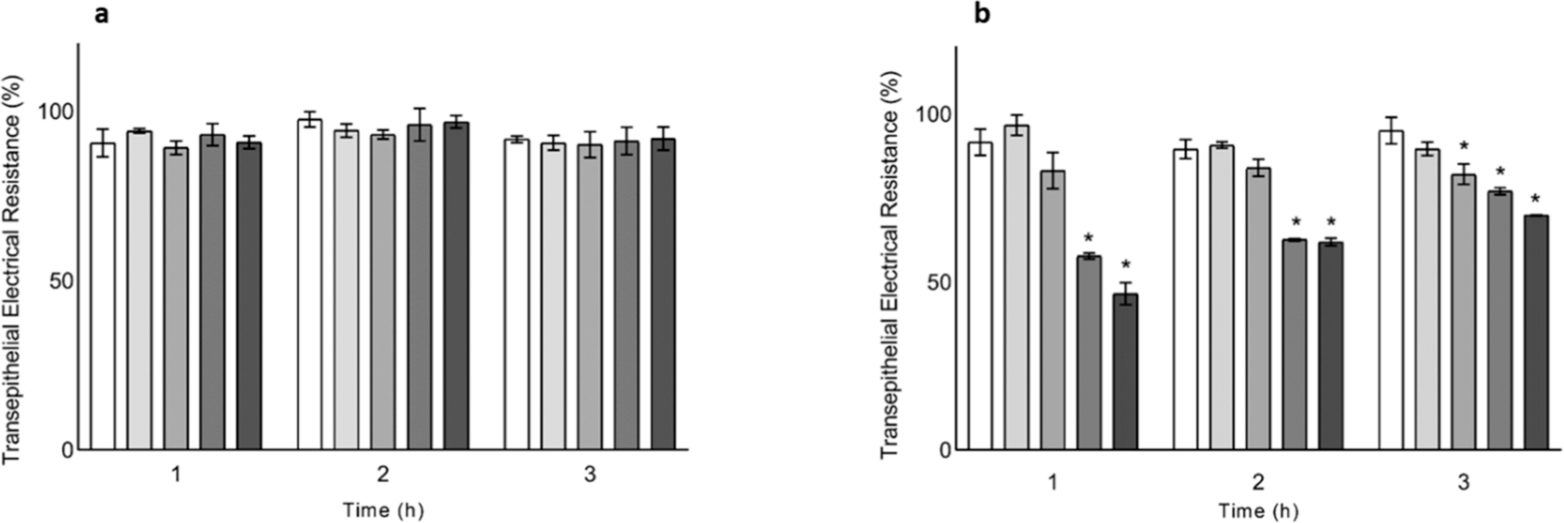
Transepithelial electrical resistance (TEER, %) (mean ± SEM) after 1, 2, and 3 h of incubation with (a) microbiota pellets and (b) supernatants from R2 (transverse colon, pH 6.3) of the BFBL simulator supplemented with Ace-K (W1 □, W2 light gray ■, W3 dark gray ■, W4 dim gray ■, W5 ■). *denotes statistically significant differences (*p* < 0.05) with respect to the control (W1).

The results on paracellular permeability support these findings. In the case of incubation with pellets, no differences were observed with respect to the stabilization period (W1) (Figure S4a). In contrast, paracellular permeability increased after incubation with the supernatants (W4 and W5), based on the basolateral Lucifer Yellow concentration, which was higher (*p* < 0.05) respect to the stabilization period (W1) (Figure S4b).

These results suggest that metabolites derived from the Ace-K-microbiota interaction, considering the results of Ace-K alone, have a negative impact on the integrity of the intestinal epithelial barrier. As shown in Figures 2 and 4, main consumption of Ace-K and loss of epithelial integrity was observed at stages W4 and W5.

## Discussion

The health-risk associated with the Ace-K metabolism by the gut microbiota is an unexplored issue. Some *in vivo* studies indicate that Ace-K is directly absorbed in the small intestine and excreted unchanged,^13^ while other studies report that Ace-K ingestion can lead to changes in the gut microbiome,^25,35^ suggesting that Ace-K is able to reach the colon. Bonatelli et al.^19^ demonstrated that certain bacteria with specific enzymatic activities could use Ace-K as a carbon source through its biotransformation to acetoacetate and sulfamate. The proposed metabolic pathway consists of the initial hydrolysis of the Ace anion, obtained after human consumption of Ace-K, by sulfatase activity, leading to the intermediate metabolite acetoacetoamide-*N*-sulfamate (ANSA), which in a second step of hydrolysis, by amidase activity, can be degraded to acetoacetate and sulfamate.

Among the bacterial taxons that showed an increase in the BFBL gut simulator reactors as the dose of Ace-K increased, we found in the shotgun data that Bacteroidales and Eubacteriales can provide arylsulfatase activities (Supporting Information File) that could be associated with the biodegradation of Ace-K.^20^ In addition, among the proteobacteria taxons that increased in a dose-dependent manner, we found that *S. wadsworthensis* could express amidase activity (EC 3.5.1.4). Other studies have highlighted the role of proteobacteria in Ace-K degradation.^36^ Furthermore, apart from amidohydrolase activity, it has also been described that *S. wadsworthensis* expresses sulfotransferase;^37^ therefore, it is possible that with this activity it might first attack the carbonyl group of the amide and then the sulfamate group via the intermediate formation of 3-(sulfamoyloxy)­crotonic acid (SOCA), giving rise to acetoacetate and sulfamate.^18^ Acetoacetate can be used intracellularly as a substrate by some bacteria for butyric acid production via the butanoyl-CoA pathway using the enzyme butyrate-acetoacetate CoA-transferase.^38^ Furthermore, Ace-K consumed by the microbiota correlated significantly with the increase in butyrate in all three colonic reactors (Figure 2), indicating that microbial Ace-K metabolism is probably involved in butyric acid production. *A. hadrus*, which expresses the butyrate-acetoacetate CoA-transferase, was found to increase with Ace-K feeding (Figure 1). In addition, *A. hadrus* and butyric acid contents correlated significantly (ρ 0.518* R1, ρ 0.521* R2, ρ 0.721** R3) in all three reactors (Figure S5), so *A. hadrus* could be contributing to the increase of butyric acid production by the utilization of acetoacetate as one of the Ace-K degradation metabolites. Moreover, members of the family Lachnospiraceae, such as the genera *Blautia*, *Coprococcus*, and *Anaerostipes*, which increased their abundances as a dose response to Ace-K (Figure 1), are the main butyrate-producing bacteria and may also be related to the observed butyrate increase (Figure 2). In addition, the metagenomic data confirmed the species found in reactors R1 and R3 of the BFBL gut simulator that possess such enzymatic activities (Figure 3) and could contribute to the microbial degradation of Ace-K. Likewise, *Intestinimonas butyriciproducens* known to produce butyrate from lysine via acetoacetate^39^ was most abundant in the R3 microbiome supplemented with Ace-K (Figure S3, Supporting Information File).

On the other hand, sulfamate could be released into the extracellular medium as a final degradation metabolite of Ace-K.^19^ This metabolite might be involved in the disruption of the epithelial integrity shown during the interaction of Ace-K-fed microbiota supernatants with Caco-2 cells (Figures 4 and S6). Results from a drug study, in which intestinal permeability was one of the parameters to be evaluated, showed that sulfamate derivates (methyl sulfamate and dimethylsulfamate) caused an increased permeability in Caco-2 cells.^40^ Furthermore, sulfamate has been reported to cause mucosal irritation.^41^ On the other hand, the increased concentration in supernatants from samples W4 and W5 of butyric acid, which is known to strengthen the barrier function,^42^ seemed to partially restore the Caco-2 epithelial integrity after 3 h of incubation (Figure 4). Nevertheless, high administration of Ace-K (150 mg/kg/day) to young C57BL/6J mice have demonstrated to cause intestinal injury with enhanced lymphocyte migration to intestinal mucosa.^35^ The gut microbiome effects and health risk associated with Ace-K consumption might be underestimated due to the widespread exposure to NNS mixtures.^43,44^

This study represents a description of compositional, functional, and metabolic changes of the child gut microbiota that follow a dose-dependent trend upon Ace-K supplementation at ascending doses within the average and high level exposure range. Furthermore, it shows the impact on the intestinal epithelial barrier derived from the Ace-K microbial metabolism. Ace-K supplementation leads to microbial changes in favor of taxa that have enzymatic activities that allow the utilization of Ace-K as a carbon source. These microbial changes respond to notable increases in *Anaerostipes*, *Coprococcus*, *Subdoligranulum*, *Blautia*, *S. wadsworthensis*, *Alistipes,* and *B. thetaiotaomicron*. On the other hand, metabolites produced from the microbial degradation of Ace-K, mainly butyric acid, also increased with dose-response effect, which in turn correlated with the Ace-K utilized by the microbiota. Some of these members of the microbiota exert enzymatic activities compatible with sulfatase and amidase activities, necessary to degrade Ace-K to obtain acetoacetate as a final metabolite, which in turn can be used to produce butyric acid via the butanoyl-CoA pathway. Regarding the epithelial barrier function, a dose-dependent decrease in the intestinal epithelial integrity occurred after metabolism of Ace-K by gut microbiota that could be caused by the release of sulfated metabolites such as sulfamate. Therefore, further experiments would be needed to elucidate the specific molecular pathways responsible for the damage of the epithelial tight-junction structures. Overall, our results suggest that the gut microbiome can metabolize Ace-K, which involves risk associated with intestinal epithelial damage. Since this study has used a wide range of Ace-K concentrations to establish dose-dependent responses, an accurate assessment of the sweetener risk is required that includes both dietary exposure and the role of the gut microbiome.

## Supplementary Material





## Data Availability

Metataxonomic and metagenomic raw sequence data are available at DIGITAL.CSIC: http://hdl.handle.net/10261/400899.

## Notes

Relative abundance of enzymatic functions involved in the microbial degradation of Ace-K, specifically sulfatases, amidases, and butyrate-acetoacetate CoA-transferase activities (XLSX)
Comparison of the alpha-diversity indexes between the microbiota supplemented with Acesulfame-K (Ace-K) and the control and the SCFA and ammonium values in the same Ace-K and control samples; relative abundance of genes conferring antibiotic resistance in the microbiota treated with Ace-K; viability of Caco-2 cells exposed to Ace-K; supernatants from the microbiota fed on Ace-K; epithelial integrity of Caco-2 cells treated with Ace-K; microbiota fed on Ace-K; correlation of *A. hadrus* abundance, butyric acid concentration, and Ace-K supplementation; and viability of Caco-2 cells exposed to sulfamic acid (PDF)
