# Supplementary material for: Human Gut Microbiome Can Degrade the Sweetener Acesulfame K with Potential Damaging Effects in the Intestinal Barrier Function
Source: J Agric Food Chem. 2026 Apr 21;74(17):13990–7. doi: 10.1021/acs.jafc.5c16498 (PMC13154174; doi:10.1021/acs.jafc.5c16498)
Supplement: Supplementary file 2 [file jf5c16498_si_002.pdf]

Supporting Information

Human gut microbiome can degrade the sweetener Acesulfame K with potential damaging effects in the intestinal barrier function

Alicia Bellanco,[a] Cristina Yépez-Notario,[a] Marta Lozano,[b] M. Carmen Martínez-Cuesta,[a] Teresa Requena*[a]

[a]Department of Food Biotechnology and Microbiology, Instituto de Investigación en Ciencias de la Alimentación (CIAL-CSIC)

[b]Department of Microbiology and Biochemistry of Dairy Products, Instituto de Productos Lácteos de Asturias (IPLA-CSIC),

*Correspondence to: Teresa Requena, t.requena@csic.es

Table S1. Values (mean ± SEM) of Shannon, Simpson and Chao1 alpha diversity indexes of the microbiota from the colonic reactors of the BFBL system (R1, R2 and R3) supplemented with Ace-K (0, 0.5, 1.5, 3 and 5 g/L) or at one week-intervals during 5 weeks with the nutrient medium (Ctrl); samples W1, W2, W3, W4 and W5, respectively.

		Shannon		Simpson		Chao1	
		**Ace-K**	**Ctrl**	**Ace-K**	**Ctrl**	**Ace-K**	**Ctrl**
**R1**	**W1**	3.4 ± 0.1	3.4 ± 0.0	0.8 ± 0.0	0.8 ± 0.0	276.2 ± 6.8	299.1 ± 11.7
	**W2**	3.5 ± 0.0	3.6 ± 0.1	0.8 ± 0.0	0.8 ± 0.0	292.4 ± 13.2	266.3 ± 21.3
	**W3**	3.7 ± 0.0	4.0 ± 0.3	0.8 ± 0.0	0.9 ± 0.0	268.1 ± 5.6	254.9 ± 38.9
	**W4**	3.7 ± 0.1	4.4 ± 0.2	0.9 ± 0.0	0.9 ± 0.0	269.4 ± 19.8	254.3 ± 37.0
	**W5**	3.6 ± 0.0	4.1 ± 0.0	0.8 ± 0.0	0.9 ± 0.0	294.2 ± 8.6	250.6 ± 6.6
**R2**	**W1**	3.4 ± 0.0	3.5 ± 0.2	0.8 ± 0.0	0.8 ± 0.0	292.4 ± 3.3	323.4 ± 29.4
	**W2**	3.7 ± 0.0	3.8 ± 0.1	0.8 ± 0.0	0.8 ± 0.0	300.8 ± 6.2	297.8 ± 25.5
	**W3**	3.8 ± 0.1	4.2 ± 0.2	0.8 ± 0.0	0.9 ± 0.0	286.4 ± 5.7	278.4 ± 20.8
	**W4**	2.3* ± 1.7	4.5 ± 0.1	0.5* ± 0.3	0.9 ± 0.0	238.4 ± 64.3	317.0 ± 16.5
	**W5**	4.0 ± 0.1	4.2 ± 0.1	0.9 ± 0.0	0.9 ± 0.0	305.1 ± 5.3	277.3 ± 8.9
**R3**	**W1**	3.6 ± 0.0	3.8 ± 0.2	0.8 ± 0.0	0.8 ± 0.0	317.0 ± 3.5	321.1 ± 23.9
	**W2**	3.7 ± 0.1	3.7 ± 0.1	0.8 ± 0.0	0.8 ± 0.0	301.6 ± 0.8	317.3 ± 40.5
	**W3**	4.0 ± 0.1	4.2 ± 0.1	0.8 ± 0.0	0.9 ± 0.0	314.3 ± 4.2	300.8 ± 15.8
	**W4**	4.0 ± 0.1	4.6 ± 0.2	0.9 ± 0.0	0.9 ± 0.0	311.0 ± 10.6	303.1 ± 23.4
	**W5**	4.1 ± 0.0	4.2 ± 0.1	0.9 ± 0.0	0.9 ± 0.0	342.7 ± 6.3	267.4 ± 12.2

* Denotes significant difference ($p<0.05$) between Ace-K and Ctrl

Table S2. Values (mean ± SEM) of the concentration (mM) of butyrate, propionate, acetate and ammonium in the colonic reactors of the BFBL system (R1, R2 and R3) supplemented with Ace-K (0, 0.5, 1.5, 3 and 5 g/L) or at one week-intervals during 5 weeks with the nutrient medium (Ctrl); samples W1, W2, W3, W4 and W5, respectively.

		Butyrate		Propionate		Acetate		Ammonium	
		**Ace-K**	**Ctrl**	**Ace-K**	**Ctrl**	**Ace-K**	**Ctrl**	**Ace-K**	**Ctrl**
**R1**	**W1**	10.6* ±0.1	15.0±0.1	12.0*±0.6	6.6 ±0.3	50.8*±1.8	37,0 ± 0,8	9,9 ± 1,2	9,7 ± 0,6
	**W2**	13.5 ±0.6	13.1±0.5	11.0*±0.5	8.3 ±0.4	43.4 ±3.5	36,4 ± 1,3	10,4 ± 0,8	10,3 ± 0,3
	**W3**	18.6* ±1.2	14.1±0.5	5.3 ±0.1	5.6 ±0.2	39.2 ±2.5	38,5 ± 0,5	10,9 ± 0,3	10,3 ± 0,4
	**W4**	19.0* ±0.9	10.5±1.7	5.7 ±0.3	5.9 ±0.2	40.0 ±0.7	36,0 ± 2,1	10,9 ± 0,3	9,4 ± 1,1
	**W5**	20.1* ±1.0	11.9±0.5	6.1 ±0.5	7.5 ±0.3	39.8 ±3.0	44,1 ± 1,8	10,6 ± 0,2	10,4 ± 0,4
**R2**	**W1**	9.1 ±0.5	10.6±0.1	14.6*± 1.1	10.3±0.6	59.6 ±3.6	63,5 ± 0,1	14,7 ± 1,0	15,1 ± 0,4
	**W2**	13.0* ±0.7	9.3 ±0.1	13.9 ±0.9	12.9±0.1	55.2 ±1.4	55,2 ± 1,3	14,7 ± 0,6	14,1 ± 0,6
	**W3**	13.6 ±0.7	11.5±0.1	9.8 ±0.6	9.7 ±0.2	48.3 ±0.7	56,1 ± 0,4	14,8 ± 0,3	14,2 ± 0,1
	**W4**	15.6* ±0.4	9.7 ±1.1	8.6 ±0.3	9.2 ±0.5	49.9 ±0.6	52,8 ± 3,2	15,4 ± 0,2	12,8 ± 1,4
	**W5**	17.2* ±0.3	8.0 ±0.3	9.3 ±0.4	10.5±0.2	49.8 ±0.4	58,5 ± 1,0	14,7 ± 0,6	13,0 ± 0,1
**R3**	**W1**	4.8 ±1.3	3.4 ±0.6	16.7*±0.3	12.9±0.7	77.6*±3.4	87,7 ± 5,3	16,4 ± 1,0	16,0 ± 0,7
	**W2**	7.4* ±0.3	1.9 ±0.3	17.3 ±0.3	16.5±0.3	77.8 ±0.9	74,5 ± 1,6	16,8 ± 0,7	14,7 ± 0,3
	**W3**	9.5* ±0.8	3.9 ±0.1	13.1 ±0.8	13.2±0.5	65.0*±1.1	74,7 ± 0,7	17,1 ± 0,5	15,4 ± 0,4
	**W4**	12.6* ±0.5	3.4 ±0.0	10.9 ±0.1	11.2±0.7	66.5 ±0.9	73,0 ± 4,8	18.5*± 0,2	14,1 ± 1,0
	**W5**	13.1* ±0.4	1.6 ±0.2	12.0 ±0.6	12.3±0.1	66.4 ±1.1	72,7 ± 0,4	16.9*± 0,6	12,9 ± 0,6

* Denotes significant difference (p<0.05) between Ace-K and Ctrl

Table S3. Relative abundance (%, average of the reactors R1 and R3) of the genes conferring antibiotic resistance from the BFBL gut simulator fed with Ace-K or not (control) at the end of experiments (W5).

Antibiotic resistance	Gen	Ace-K	Control
Glycopeptide antibiotic	*van*	0.040	0.062
Fluoroquinolone	*adeF*	0.013	0.022
Tetracycline	*tet*	0.017	0.017
Sulfonamide	*sul2*	0.000	0.005
Lincosamide	*lsaA*	0.002	0.000
Fluoroquinolone efflux pump	*emr*	0.001	0.004
Macrolide efflux pump	*evg*	0.001	0.001

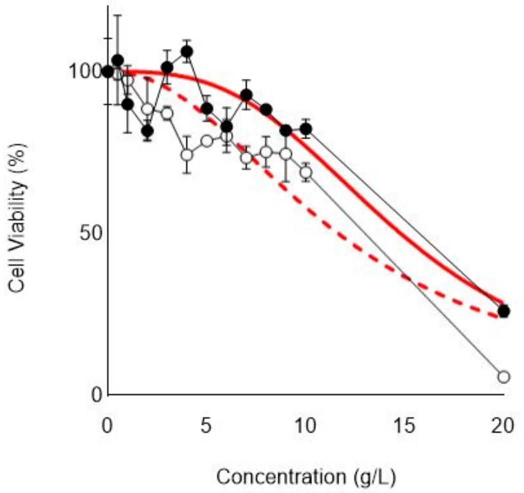

Figure S1. Values (%) of Caco-2 cells viability (mean +-SEM) after 24 (●) and 48 h (○) of incubation with acesulfame K at increasing concentrations, and its IC_{50} curve at 24 (---) and 48h (- - -).

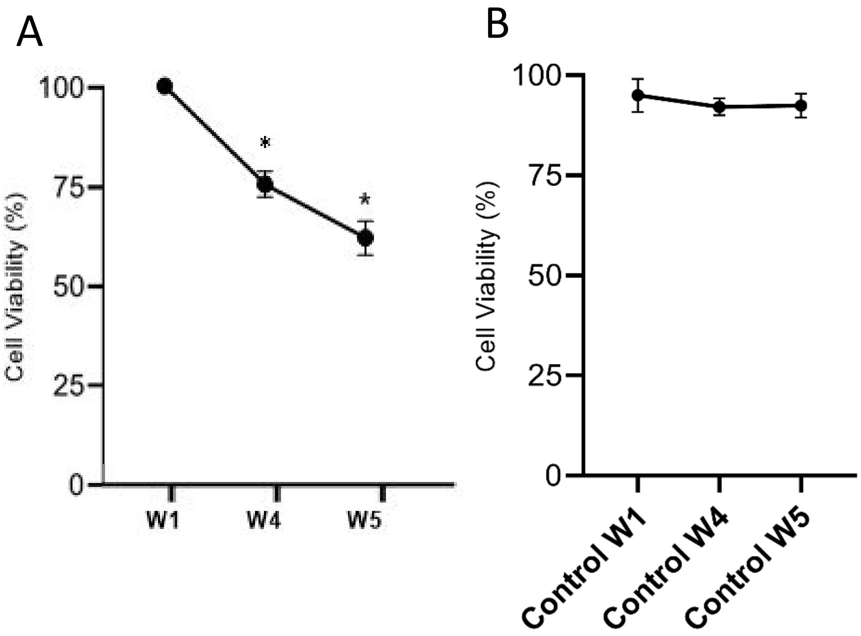

Figure S2. Values (%) of Caco-2 cells viability (mean +-SEM) after 24 h of incubation with supernatants from the BFBL gut simulator (R2) supplemented with acesulfame K (A) and during 5 weeks with the nutrient medium (B). *denotes statistically significant differences ($p<0.05$) with respect to the stabilization week (W1).

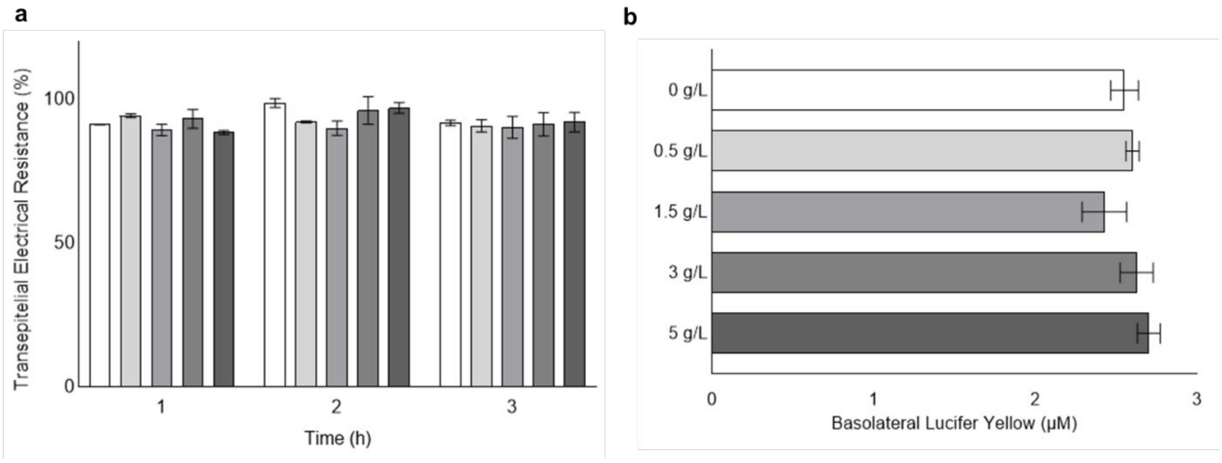

Figure S3. (a) Transepithelial electrical resistance (%) (mean ± SEM) after 1, 2 and 3 h incubation with Ace-K (0 □, 0.5 □, 1.5 □, 3 □, 5 ■ g/L) and (b) basolateral concentration (µM) of Lucifer Yellow (mean ± SEM) after 3 h incubation with Ace-K at the same concentrations.

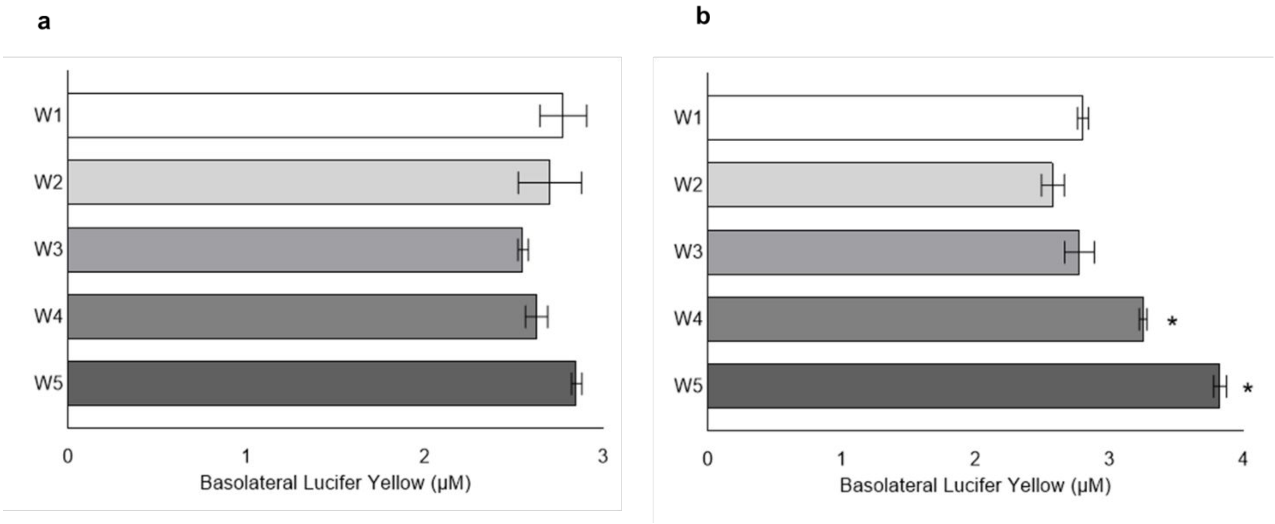

Figure S4. Basolateral concentration (uM) of Lucifer Yellow (mean ± SEM) after 3 h incubation with (a) pellets and (b) supernatants from the BFBL simulator fed with Ace-K (W1 ☐, W2 ▩, W3 ▩, W4 ▩, W5 ▩). * denotes statistically significant differences ($p<0.05$) with respect to the control

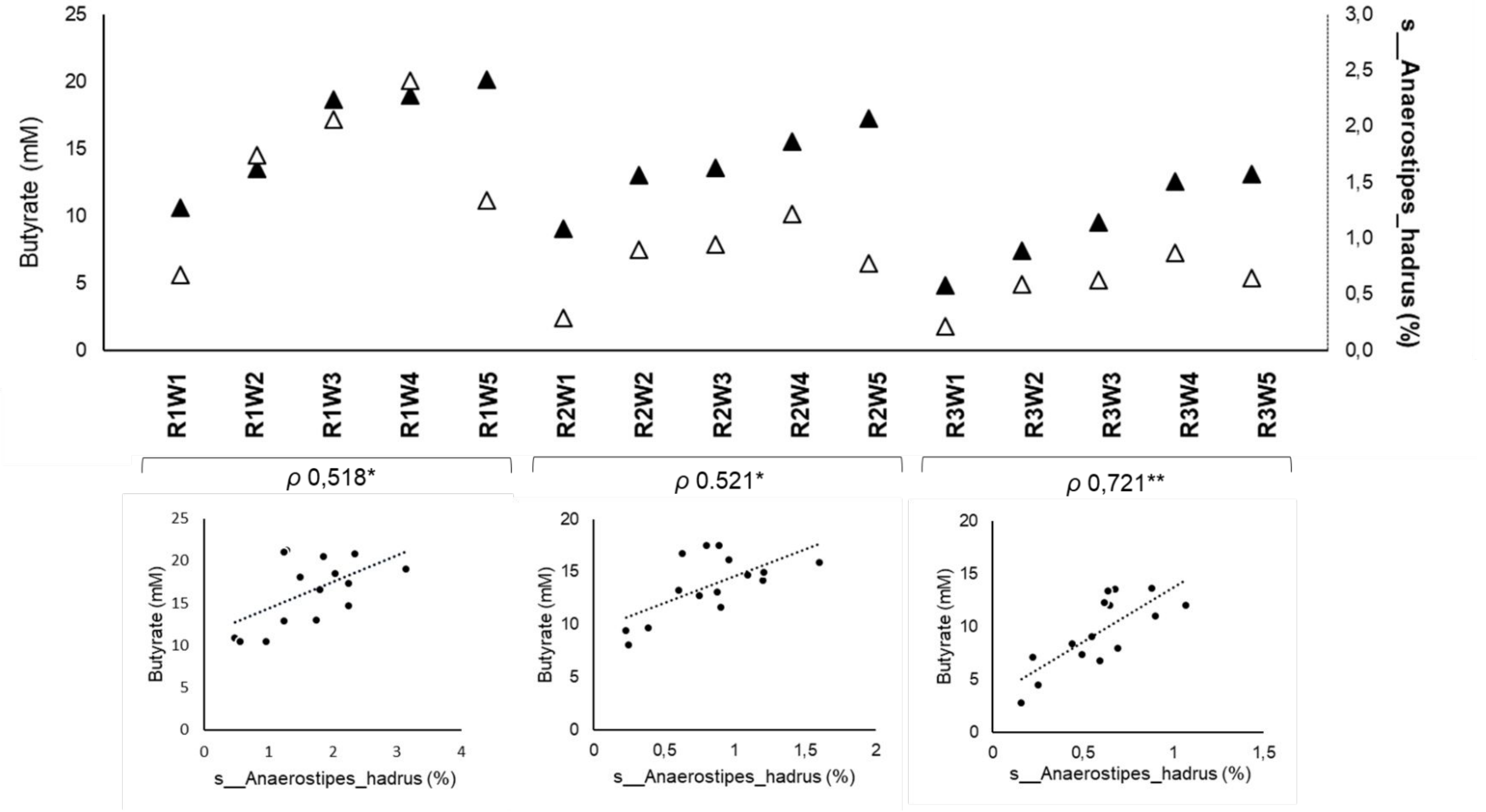

Figure S5. Relative abundance of *Anaerostipes hadrus* (OTU %) (△) and butyric acid concentration (mM) (▲), and their correlation, in the colonic reactors of the BFBL simulator (R1, R2 and R3) fed with increasing doses of Acesulfame-K (W1, W2, W3, W4 and W5). Spearman's Rho (ρ) indicates correlation between the relative abundance of *A. hadrus* (OTU %) and produced butyrate (mM) by Ace-K-fed microbiota. * and ** denote significant correlation (p<0.5 or p<0.1, respectively).

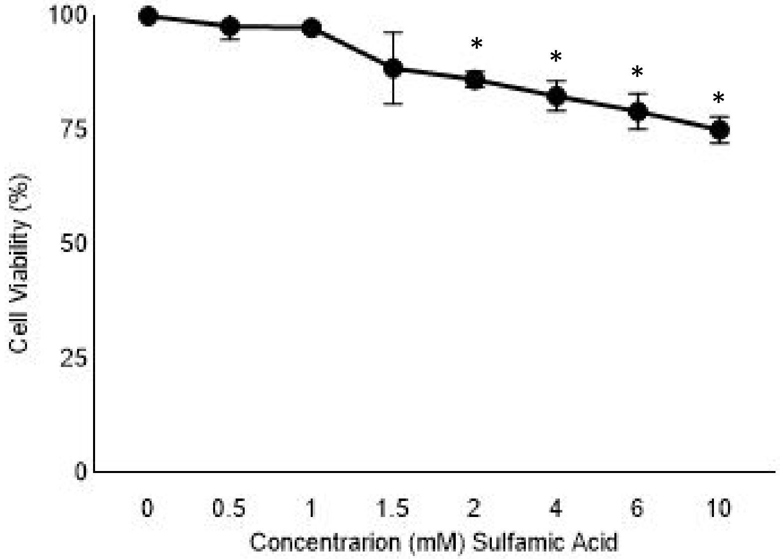

Figure S6. Values (%) of Caco-2 cells viability (mean +-SEM) after 24h of incubation with sulfamic acid at increasing concentrations. * denotes statistically significant differences ($p<0.05$) with respect to the control (0 g/L).
